# Extremely Acidic Eukaryotic (Micro) Organisms: Life in Acid Mine Drainage Polluted Environments—Mini-Review

**DOI:** 10.3390/ijerph19010376

**Published:** 2021-12-30

**Authors:** Ana Teresa Luís, Francisco Córdoba, Catarina Antunes, Raul Loayza-Muro, José Antonio Grande, Bruna Silva, Jesus Diaz-Curiel, Eduardo Ferreira da Silva

**Affiliations:** 1 GeoBioTec Research Unit, Department of Geosciences, University of Aveiro, 3810-193 Aveiro, Portugal; catarinaantunes98@ua.pt (C.A.); bruna.ssilva@ua.pt (B.S.); eafsilva@ua.pt (E.F.d.S.); 2 Department of Water, Mining and Environment, Scientific and Technological Center of Huelva, University of Huelva, 21007 Huelva, Spain; grangil@uhu.es; 3 Department of Integrated Sciences, Faculty of Experimental Sciences, University of Huelva, 21007 Huelva, Spain; fcordoba@uhu.es; 4 Laboratório de Ecotoxicología, Facultad de Ciencias y Filosofiia, Universidad Peruana Cayetano Heredia, Av. Honorio Delgado 430, Lima 15102, Peru; raul.loayza@upch.pe; 5 Sustainable Mining Engineering Research Group, Department of Mining, Mechanic, Energetic and Construction Engineering, Higher Technical School of Engineering, University of Huelva, 21007 Huelva, Spain; 6 Escuela Técnica Superior Ingenieros de Minas, Rios Rosas 21, 28003 Madrid, Spain; j.diazcuriel@upm.es

**Keywords:** AMD (Acid Mine Drainage), metal mining, extremophilic organism, green algae, micro-macroinvertebrates, fungi, Rotifera, *Euglena*, protozoa

## Abstract

Acid Mine Drainage (AMD) results from sulfide oxidation, which incorporates hydrogen ions, sulfate, and metals/metalloids into the aquatic environment, allowing fixation, bioaccumulation and biomagnification of pollutants in the aquatic food chain. Acidic leachates from waste rock dams from pyritic and (to a lesser extent) coal mining are the main foci of Acid Mine Drainage (AMD) production. When AMD is incorporated into rivers, notable changes in water hydro-geochemistry and biota are observed. There is a high interest in the biodiversity of this type of extreme environments for several reasons. Studies indicate that extreme acid environments may reflect early Earth conditions, and are thus, suitable for astrobiological experiments as acidophilic microorganisms survive on the sulfates and iron oxides in AMD-contaminated waters/sediments, an analogous environment to Mars; other reasons are related to the biotechnological potential of extremophiles. In addition, AMD is responsible for decreasing the diversity and abundance of different taxa, as well as for selecting the most well-adapted species to these toxic conditions. Acidophilic and acidotolerant eukaryotic microorganisms are mostly composed by algae (diatoms and unicellular and filamentous algae), protozoa, fungi and fungi-like protists, and unsegmented pseudocoelomata animals such as Rotifera and micro-macroinvertebrates. In this work, a literature review summarizing the most recent studies on eukaryotic organisms and micro-organisms in Acid Mine Drainage-affected environments is elaborated.

## 1. Introduction

Acid Mine Drainage (AMD) is one of the main hydrological and geochemical problems derived from anthropogenic influence on the geosphere, which affects many countries with intense mining activities [1,2].

AMD is produced when sulfide-bearing materials suffer direct oxidation, which is then spread by the indirect oxidation of ferric ions. Chemical oxidation processes can be biologically “catalyzed” by some bacteria [3]. Along with pyrite reactions [4], many other associated reactions can be produced by the remaining metals which, in the form of sulfur, appear along with pyrite. As a result of these reactions and due to the very acidic waters, numerous soluble contaminating elements, are stored on pyrite surfaces and transported by inland streams. This process is affected by numerous factors including the type, abundance and distribution of sulfides and minerals with neutralizing capacity as well as oxygen concentration, humidity, temperature, exposed pyrite surface area, types of bacteria, etc. [5]. When transported to inland streams, AMD can contaminate surfaces, ground sediments and soils, as a consequence of its very low pH as well as by a high content of sulfates and heavy metals in water and a high metallic content in sediments [6,7].

Both active and abandoned mines are major sources of AMD [8], which is not only generated in sulfide mines, e.g., the Iberian Pyrite Belt, but also to a lesser extent in coal mines, e.g., the Northern Appalachian Coalfield. In the nearest streams, this could result in species loss and significant structural changes to freshwater organisms [9] and loss of species richness [10] and macroinvertebrate abundance [11].

AMD is responsible for the disappearance of several species of algae and diatoms, such as the *Cyclotella* and *Fragilaria* genera [12], and for loss of diatomic diversity in impacted sites [13] dominated by typical species of acidic waters [14,15], for example *Pinnularia acoricola* and *Eunotia exigua*. At the community level, the highest metal concentrations (along with pH < 3 and high Eh potential) implicate the lowest diversity [16], while at the individual level changes in frustule morphology are observed [15,17]. With respect to filamentous green algae, acidophilic species of the *Mougeotia* and *Klebsormidium* genus are abundant in AMD streams [18,19], as are other unicellular algae such as *Chlamydomonas* or *Euglena*, which may be very abundant in such environment, along with Protozoa and some multicellular protists. In relation to the impact of AMD on macroinvertebrates, there is evidence of a high impact on density and taxa richness [20] as well as a change in the shape of the food pyramid [21]. In places affected by AMD, species of macroinvertebrates can be found, which are tolerant to these environments, for example, chironomids; on the other hand, the most sensitive species, for example, flies, are excluded as a result of the low pH and high concentrations of metals [22].

Therefore, the main objective of this review is to summarize the scientific literature related to AMD production and its effects on eukaryotic organisms thriving in the water or sediments of streams and rivers, focusing on the following subjects: Acid Mine Drainage and the impact of AMD on algae, Protozoa, fungi and yeast as well as on micro- and macroinvertebrates.

## 2. Acid Mine Drainage

Around the world, there are mines that have been abandoned and pose a long-term threat to aquatic ecosystems due to the continuous or intermittent flow of acidic drainage water containing high concentrations of various heavy metals [23]. AMD is predominantly caused when sulfide minerals present in metallic ores, coal beds, or the strata overlying and underlying the coal are exposed to weathering causing oxidation [24,25], which later on is propagated through indirect oxidation by ferric ions produced mainly by chemolithotrophic bacteria [26,27]. Chemical reactions such as hydrolysis and oxidation can transform sulfide minerals into sulfuric acid, decreasing the pH of water at active or abandoned mine sites [28]. Mine facilities, tailings and waste rocks left in these sites are major contamination sources of AMD (Okabayashi et al., 2005). Metal-bearing minerals are abundant in finely-ground mine tailings or fine particles of by-product from mining activities [28]. Tailings with 5% pyrite and arsenopyrite are high enough to produce AMD [29].

In mine waste materials containing sulfide minerals (pyrite, galena, sphalerite and arsenopyrite), AMD is produced due to natural oxidation reactions involving the exposed sulfides, air, water, and soil microorganisms [30]. An AMD with high potential of reactivity promotes the dissolution of the bedrock, mobilising heavy metals that will change the stream water quality and the groundwater system [30]. The Iberian Pyrite Belt (IPB) has one of the world’s largest concentrations of sulfide deposits, running from Lousal, Portugal to Aznalcóllar, Spain [31]. In Andalusia, southwestern Spain), thousands of years of mining in the IPB have resulted in enormous metal wastes [32] that severely degrade the environment [4,33]. The IPB zone has massive sulfide reserves of around 1700 Mt that are distributed across more than 50 massive sulfide deposits [34]. The Spanish side of the IPB has 88 mines [4,35], most generating AMD, in an area with more than 4000 ha of waste rock and tailings [31]. The Odiel River Basin is a well-known fluvial system in a catastrophic ecological situation due to AMD affecting 37% of its drainage network length [33]. The Odiel River is affected from its upper section to the Huelva estuary. In fact, the Ria de Huelva is one of the most heavily metal-contaminated estuaries in the world as a result of AMD from the IPB mines [36].

Coal originates in the burial of organic matter in swamps, and pyrite is also formed in these environments. One of the major sources of water pollution in and around both active and abandoned coal mines is AMD [37]. This becomes even more severe with marine influence in coal deposits, due to the presence of additional framboidal pyrite [38]. Pyrite in coal oxidizes when exposed to air and water, producing Fe (III) and H_2_SO_4_ [39]. Fe (II) ions are oxidized, forming Fe oxide and producing H^+^ ions, lowering the pH of the water and making it corrosive [39]. The Northern Appalachian Coalfield in the eastern US has a historical legacy of coal mining [40] and represents one third of the abandoned mine-related problems in the country [41]. The Witbank Coalfield, located in the headwaters of the Olifants River in Mpumalanga Province, South Africa is dominated by past and present coal mining, and AMD from these mines results in both a low pH and high total dissolved solids in this river, which then flows through areas of intensive agriculture [42].

Small-scale gradients of pH and metals within such systems can be useful as field model systems to study the biological effects of acid and metal pollution [23]. The information gained is very important because it might be useful to develop bioassessment methods. The mitigation of not only the physical, chemical and biological, but also the socioeconomic impacts of AMD is one of the major challenges faced by the mining industry worldwide, and many countries have been investing in the development of efficient treatment methods for wastewater from mining.

## 3. Eukaryotic Organisms in AMD-Polluted Extreme Environments

### 3.1. Diatoms

The diatoms are one of the most effective ecological indicators [14,15,16,17,20,31,43] in AMD-contaminated environments, due to their ubiquity in aquatic habitats [44] and high effectiveness for assessing aquatic health [45]. Thus, they are good indicators of pH changes and very abundant in environments impacted by low pH [46]. Diatoms respond to chemical stress at community and individual levels. At a community level, the highest metal concentrations (i.e., Fe: 6 g/L, Zn 1.7 g/L, Cu 347 mg/L, Cd 3.5 mg/L, Ni 3 mg/L, Mn 0.3 mg/L) and low pH (i.e., 2.0-4.5) result in low diatom diversity (Shannon–Winer diversity index < 2.2 on a 5-point scale) [14,15,16], and the species change to more acidophilic or acidobiontic varieties better-prepared to endure these harsh conditions. This decrease in species richness has been observed in many works [13,15,16,20,31,43,47,48], and is more prominent for diatoms than for macroinvertebrates [23].

**Figure 1:**
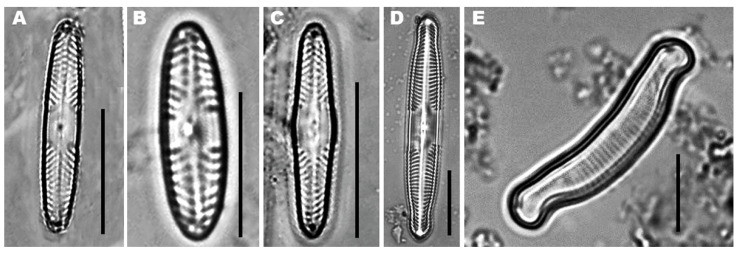
(**A**) *Pinnularia acidophila*, (**B**) *Pinnularia acoricola*, (**C**) *Pinnularia aljustrelica*, (**D**) *Pinnularia subcapitata* and (**E**) *Eunotia exigua*.

The dominant and typical species in acidic waters are *Pinnularia acoricola, Pinnularia acidophila*, *Pinnularia aljustrelica*, *Eunotia exigua* (Figure 1) and *Nitzschia hantzschiana* [13,14,15,31]. The three *Pinnularia* species found in the impacted sites; *P. aljustrelica* is the most abundant due to its capacity to survive a very low pH, i.e., 1.9–4.2 [15,49]. *Achnanthidium minutissimum* is a difficult species, able to tolerate different environmental conditions and usually the only *Achnanthidium* species reported in AMD polluted streams [50], being abundant in a wide variety of habitats and environmental conditions [51]. However, *A. minutissimum* can also appear in unimpacted sites, being the dominant species in less-impacted sites [14]. It is considered to generally be the first taxon to colonize different habitats (e.g., rocks, sediments) [52], and has the ability to invade open areas following changes in environmental conditions [53].

Some of the other ‘true inhabitants of highly acidic waters’ [20] include *Nitzschia capitellata*, *Nitzschia subcapitellata* and *Pinnularia subcapitata* [54]. In sites with pH below 4.5, *Eunotia exigua*, *Nitzschia* cf. *thermalis*, *Pinnularia acidophila*, *Pinnularia acoricola*, *Pinnularia subcapitata* and *Pinnularia aljustrelica* can appear [31]. This fact is supported by other authors who have found these species under similar environmental conditions [16,46,49,55].The *Pinnularia* and *Achnanthes* genera (especially *Pinnularia*) are often the most frequent in impaired sites [56], implying that these genera are tolerant to AMD [22,57] and making them particularly useful as bioindicators of low pH [58].

In the Lousal and Aljustrel mining areas located in the Portuguese part of the IPB, the species found (in descending order of dominance) include *Brachysira vitrea*, *Eunotia exigua* and *Pinnularia* c.f. *acidophila* (Figure 1). In the Aljustrel mining area, with sulfated high to extreme metal/metalloid concentrations and low pH waters, *P. aljustrelica*, *E. exigua* (Figure 1) and *Nitzschia* aff. *hantzschiana* are the dominant species [15]. However, *E. exigua* is an acidobiontic taxon, and is the most widespread species in AMD-contaminated streams such as the Río Tinto [16,31,59,60,61] and the Aljustrel streams [14,15,17,55] (Table 1).

Metals lower biodiversity in several important ways. Diatoms have developed mechanisms such as biotransformation, biomineralization, bioaccumulation and biosorption to cope with heavy metal toxicity [62]; nevertheless, pollution-tolerant and pollution-sensitive diatoms have different responses to metal pollution [63]. When exposed to metals, community size can be impaired through reduction of cell number, selection for smaller species, and decrease in cell size within a given species [17,64,65,66]; diatom growth can be delayed or inhibited, therefore reducing diatom biomass [67] and decreasing the rates of survival and growth. Diatoms are able to sequestrate large quantities of metals from waters [68]. The most common taxa presenting abnormal valves due to metals/pH or metal-pH combination are *Fragilaria capucina* [69], *Fragilaria rumpens* and *A. minutissimum* [69] and *Eunotia exigua* [15].

Thus, the observed differences in diatom community structure result from the combined action of low pH and highly soluble heavy metals [54,70]. Diatoms can also be susceptible at the individual level showing changes in frustule morphology [17]. The resistance of *A. minutissimum* to metals is still under discussion, with contradictory results in the literature. It is usually considered an indicator of metal pollution [71], although it could also indicate good general water quality [72].

### 3.2. Unicellular and Filamentous Green Algae

Although AMD environments are not appetizing to many species, some genera of unicellular and filamentous green algae can adapt and survive; among these are species from the unicellular genera *Chlamydomonas*, *Chlorella*, *Cyanidium*, *Dunaliella*, *Euglena* [73,74] and from the filamentous genera *Klesormidium*, *Microspora*, *Mougeotia*, *Ulothrix*, *Stigeoclomium*, *Zygnema* and *Microthammion*. The genera *Mougeotia*, *Ulothrix*, *Chlamydomonas*, *Chara* and *Nitella* are typical of these environments; however, they may not be as abundant as diatoms [75,76,77].

*Cyanidium* is a red algae genus, or rhodophite. It has been observed at pH 1.2–1.8 in waters close to the Rio Tinto mines. *Dunaliella*, *Chlamydomonas* and *Chlorella* are unicellular green algae from the Chlorophyceae family. Both *Chlamydomonas* and *Dunaliella* may be motile, with the presence of flagella. Curiously, *Dunaliella* has no cell wall. *Chlamydomonas acidophila* is the most abundant species in acid waters, showing a high tolerance to copper and other heavy metals [78,79] *Euglena mutabilis* is abundant in shallow waters and easily forms large tufts that can look like filamentous algae. Oxygen bubbles are frequently observed in some places where *Euglena* thrives. All microalgae contribute to enhanced oxygen production (up to 200% saturation) and organic carbon, which reduces the oligotrophic conditions of AMD-polluted waters and increases the oxidative activity of aerobic chemoautolithotrophic bacteria and heterotrophic bacteria [80].

The acidophilic species of the *Mougeotia* genus can survive in the AMD environment, in waters with a pH of 2.9–4.1 [18]. The abundance and distribution of *Klebsormidium* sp. in AMD affected waters makes this species a good ecological indicator of this type of contamination, and *Klebsormidium*-dominated algal mats are particularly good indicators of high iron concentrations in water [81]. Additionally, *Mougeotia*, can be abundant in AMD streams [19,81], possibly because of strong competition for low DIC (dissolved inorganic carbon) in acidic environments [82]. The genus *Klebsormidium* is known to be metal resistant, and is been related with metal-rich polluted waters. *K. subtile*, *K. rivulare*, *K. flaccidium* and *K. acidophilum* are other species related with AMD-contaminated environments [19,51,77]. *Chlamydomonas* sp. shows tolerance in a wide range of physical and chemical conditions in a lake contaminated by AMD, being consistently present [83].

The *Microspora* genus is very abundant in mines with high levels of metal pollution, and is considered by [84] as a good bioindicator. The *Ulothrix* genus, on the other hand, is predominant in biofilms from AMD-contaminated sites, having a great capacity to recover Cu and As.

**Figure 2:**
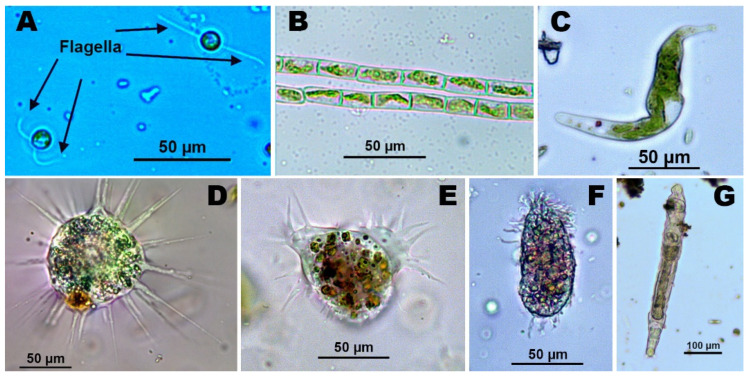
(**A**) Unicellular algae (*Chlamydomonas acidophila*), (**B**) Filamentous algae (*Klebsormidium* sp.), (**C**) *Euglena mutabilis*, (**D**) Protozoo Heliozoa, (**E**) Protozoo Ciliata, (**F**) Amoeba, (**G**) Rotífer.

Several of the microorganisms described above are represented in Figure 2.

### 3.3. Protozoa, Fungi and Yeasts in AMD-Polluted Waters

In AMD-polluted waters, several groups of heterotrophic protists may be observed. The main groups include protozoa: ciliates such as *Urotricha* and *Oxytricha*, flagellates such as *Bodo* and *Ochromonas*, amoebas such as *Actinophyrs* and *Naegleria*, etc., and heliozoa. In acidic waters, these genera play an essential role in nutrient recycling in spite of their oligotrophic characteristics [85].

**Figure 3:**
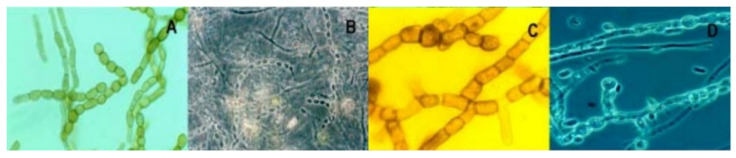
(**A**) *Scytalidium thermophilum*, (**B**) *Possible Acremonium* sp., (**C**) *Scytalidium acidophilum*, (**D**) *Lecythophora hoffmannii*. Photos from [89]. Photos adapted from ref. [89].

Fungi are more acidotolerant than acidophilic, although some filamentous fungi, such as as *Acontium*, *Cephalosporium* and the yeast *Trichosporon*, are able to growth up to pH 0 [85]. In [86], a wide variety of filamentous fungi are described, including *Scytalidium*, *Bahusakala*, *Phoma*, *Heteroconium*, and even *Penicillium* and diverse ascomycetes and zygomycetes. In addition to their role as components of an acidic river ecosystem, fungi play an important role in the biomineralization of iron and the accumulation of intracellular deposits of toxic metals [87,88]; see examples below (Figure 3).

### 3.4. The Impact of AMD on Micro-Macroinvertebrates

AMD represents an extremely stressful and long-term source of pollution due to the anthropogenic disturbance of geological layers. Characteristic low pH and high metal concentrations have been highlighted as the main drivers of micro- (<500 µm length) and macroinvertebrate (>500 µm, length) diversity and community composition in streams affected by AMD [11,20,90], while acidification may induce an increase in the bioaccumulation of metals in insect larvae with consequences for the food chain and aquatic fauna [91]. The main microinvertebrates observed in these waters are from phylum Rotifera, considered pseudocoelomate “animals” [89,92,93].

Variation in macroinvertebrate assemblages and densities has also shown a strong relationship with other water chemistry variables in addition to metals, such as dissolved oxygen and conductivity, inducing a clear shift from metal-sensitive (e.g., Ephemeroptera, Plecoptera and Trichoptera) to metal-tolerant (Diptera, Coleoptera and Collembolla) taxa [94,95,96,97,98]. The order Ephemeroptera is a group highly sensitive to metals; however, some species, such as *Baetis rhodani* and *Caenis* cf. *luctuosa*, exhibit tolerance to these contaminants [99,100].

Among metal-tolerant taxa, Chironomidae (Diptera) assemblages often represent a significant portion of the sediment-dwelling fauna at deteriorated sites, and are hence especially useful as bioindicators and for sediment quality assessment [101,102,103]. Chironomid species have been found in acidified metal-polluted temperate [103,104], tropical and high-altitude streams [96,105] as well as unpolluted glacier-elevated water streams [106]. Species of *Chironomus* may have physiological adaptations responsible for such tolerance, as those species coming from contaminated points are able to adjust their body metals concentration when compared to other species [97]. Chironomids from elevated altitudes and metal-contaminated sites contain more melanin than species from reference sites at lower altitudes [107]. This fact highlights the importance of melanin in chironomids as a UV-B radiation protector and metal chelator. In addition, genetic adaptation has been found to be a metal tolerance tool in *Chironomus* species from highly contaminated environments [108,109,110]. In [97], it was found that only one tolerant strain of chironomids was able to survive in the most metal-rich points in the Andes, which indicates that tolerance could have been developed as an answer to naturally existing acid and metal-rich environments, and thus may have preceded human-influenced alterations due to mining activity. The adaptation of this unique chironomid species to very large metal values may have come with direct costs, as represented by smaller specimens in comparison to those from species in similar reference streams, in the form of reallocation of energy towards resistance tools such as metal-binding metallothioneins. Melanin production or cuticle sclerotization in chironomids [107] may convey a trade-off evidenced as reduced growth [111].

Chironomids are used as potential biomonitors at different organizational levels in order to indicate the biological effects of metal pollution. At the cytological level, genotoxic damage produces micronuclei in the structure of salivary gland chromosomes of larvae of *Chironomus acidophilus* in a river with high concentrations of Cu, Fe, Mn and Zn [112]. At the organism level, cause–effect relationships between morphological abnormalities such as deformities of the mouthparts and metal-rich stream sediments have been demonstrated in both, laboratory [113,114] and field [115,116,117,118]. This was the case for *Chironomus tentans* larvae, where fused, split, missing, extra and abnormally-shaped teeth on the mandible were associated with different metal levels [119].

**Figure 4:**
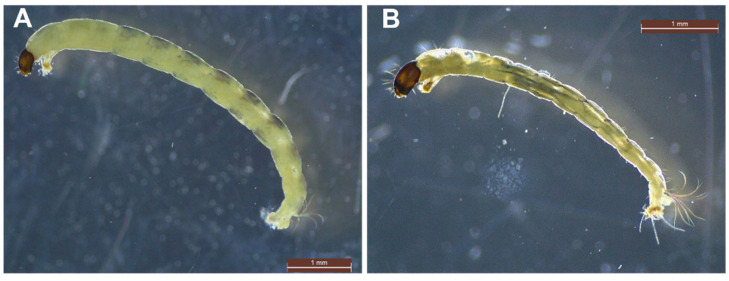
Microphotographs of individuals from family Chironomidae: (**A**) *Cricotopus* sp., (**B**) subfamily Orthocladinae.

Most taxa within the Chironomidae (Figure 4) are collector-filterers and collector-gatherers while a few (e.g., *Cryptochironomus* sp., *Endochironomus* spp., *Glyptotendipes* spp., *Polypedilum* spp. and *Chironomus* spp.) are predatory on oligochaetes in AMD-contaminated sites [100], which indicates that these group show different ecological response patterns to AMD [103]. Moreover, shredder-climbers can be the dominant group at impacted sites and could be more adaptive in AMD affected streams than other groups, as Fe-loving bacteria growing on leaves coated with Fe hydroxide become an option as a food resource [120].

In general, stress conditions may benefit the increase of secondary consumers, changing, considerably, the food chain shape [103]. This phenomenon has been described for macroinvertebrates from AMD impacted streams [100] and implies major shifts in resource utilisation, possibly reducing the number of trophic levels and consequently simplifying the food web. While these ecological processes still need further analysis in AMD environments, they can explain the use of Tanypodinae as bioindicators. This is because AMD leads to a significant change in the community structure of chironomid larvae. On the other hand, the taxonomic richness within the Chironomidae remains stable in acid mine drainage because the loss of sensitive species is compensated for by tolerant species [100]. Sites with severe AMD have a significant decrease in abundance of stationary collector-filterer prey (primarily Hydropsychidae, caddisflies that occur in high densities), showing that both the diversity and abundance of macroinvertebrate prey decreases as AMD impact increases [121]. Thus, AMD contamination sites can have high biodiversity because of high tolerant species richness, as well as considerable variability in metal tolerance among macroinvertebrate taxa and species (Byrne et al., 2012). When compared to reference sites, the functional diversity of macroinvertebrates is lessened, and their functional structure is much simpler [122].

## 4. Conclusions

This research study sought to put in evidence the importance of extremophile organisms in AMD-affected environments. This mini-review summarizes the eukaryotic groups inhabiting these environments. AMD affects the organisms inhabiting the water and sediment substrates, which are subjected to high concentrations of metals and sulfates along with low pH. AMD-provoked changes in the ecological environment at both the community level and the individual level are responsible for species disappearance and the loss of diversity and abundance. Only those organisms capable of developing adaptation mechanisms to these extreme conditions survive and succeed [123].

Further research in this area is crucial in order to minimize the evident environmental consequences of mining exploration through the centuries, with bio- and eco-friendly solutions having particular appeal.

New applications using these extreme organisms in biotechnology and astrobiology studies are the main reason for their study at present. An example is a recent study of the Tintillo River [124], contaminated by Río Tinto in Huelva, Spain, where bacterial filaments and diatoms are capable of forming iron stromatolites as laminated sedimentar structures. Furthermore, the active biosorption and bioleaching of sulfur are suggested by the black and white coloration of microbial filaments inside these stromatolites. AMD systems are hazardous to physical, chemical, and biological agents; however, they also provide valuable biogeochemical information which can aid in inferring past geochemical conditions on Earth, and perhaps even other planets such as Mars.

## Figures and Tables

**Table 1:** Diatom species with pH and metal concentrations (mg/L), pH tolerance range and optimum pH.

Species Name	pH Tolerance Range	Optimum pH	Metal Concentrations
*Pinnularia aljustrelica*	2.0–5.0	2.0–3.0	Fe 1300 to 6000 Cu 230–350 Zn 118–170
*Pinnularia acidophila*	2.0–4.5	2.0–2.2	
*Pinnularia acoricola*	2.0–6.0	2.0–3.0	
*Nitzschia thermalis*	2.0–7.0	3.0	
*Nitzschia hantzschiana*	2.0–6.8	2.0–2.2	
*Eunotia exigua*	3.0–5.0	3.0	Similar metal concentrations as above, but species valves are morphologically affected by metals (teratologies)
*Brachysira vitrea*	4.5–7.5	4.8	Fe 1100 Zn 0.30 Cu 0.64
